# Structure of human cytomegalovirus virion reveals host tRNA binding to capsid-associated tegument protein pp150

**DOI:** 10.1038/s41467-021-25791-1

**Published:** 2021-09-17

**Authors:** Yun-Tao Liu, David Strugatsky, Wei Liu, Z. Hong Zhou

**Affiliations:** 1 California NanoSystems Institute, University of California, Los Angeles, Los Angeles, CA 90095 USA; 2 Department of Microbiology, Immunology and Molecular Genetics, University of California, Los Angeles, Los Angeles, CA 90095 USA; 3 Center for Integrative Imaging, Hefei National Laboratory for Physical Sciences at the Microscale, and School of Life Sciences, University of Science and Technology of China, Hefei, China

**Keywords:** Herpes virus, Non-coding RNAs, Cryoelectron microscopy

## Abstract

Under the Baltimore nucleic acid-based virus classification scheme, the herpesvirus human cytomegalovirus (HCMV) is a Class I virus, meaning that it contains a double-stranded DNA genome—and no RNA. Here, we report sub-particle cryoEM reconstructions of HCMV virions at 2.9 Å resolution revealing structures resembling non-coding transfer RNAs (tRNAs) associated with the virion’s capsid-bound tegument protein, pp150. Through deep sequencing, we show that these RNA sequences match human tRNAs, and we built atomic models using the most abundant tRNA species. Based on our models, tRNA recruitment is mediated by the electrostatic interactions between tRNA phosphate groups and the helix-loop-helix motif of HCMV pp150. The specificity of these interactions may explain the absence of such tRNA densities in murine cytomegalovirus and other human herpesviruses.

## Abstract (web summary)

Here, cryo-EM reconstructions of human cytomegalovirus (HCMV) virions reveal host tRNAs associated with the virion’s capsid-bound tegument protein, pp150. tRNA recruitment is mediated by the interactions specific for HCMV only, suggesting the explanation for the absence of such tRNA densities in related herpesviruses.

## Introduction

Under the Baltimore nucleic acid-based virus classification scheme, herpesviruses belong to Class I viruses, meaning that their virion contains a double-stranded DNA genome—and no RNA. Biochemical analyses have shown that members of all three subfamilies of herpesviruses could package viral RNA^1–7^ in their virions. For human cytomegalovirus (HCMV), a member of the β-herpesvirus subfamily of the *Herpesviridae*, even cellular RNA molecules have been shown to be associated within its virion non-specifically^1,5^. However, none of these RNA molecules has been visualized structurally to date. Therefore, the exact locations, atomic structures, and molecular interactions of RNA molecules within these DNA viruses remain a mystery.

Here, we show tRNA-like structures associated with the capsid-associated tegument protein layer using sub-particle reconstructions with defocus refinement method. Through deep sequencing and atomic modeling, we show that host tRNA binds HCMV-specific tegument protein, pp150. Such RNA structures are absent from fellow β-herpesviruses murine cytomegalovirus (MCMV) and human herpesvirus 6B (HHV6B), suggesting HCMV-specific roles of the recruited host tRNA molecules.

## Results

### Visualization of RNA structures in HCMV tegument

**Fig. 1:**
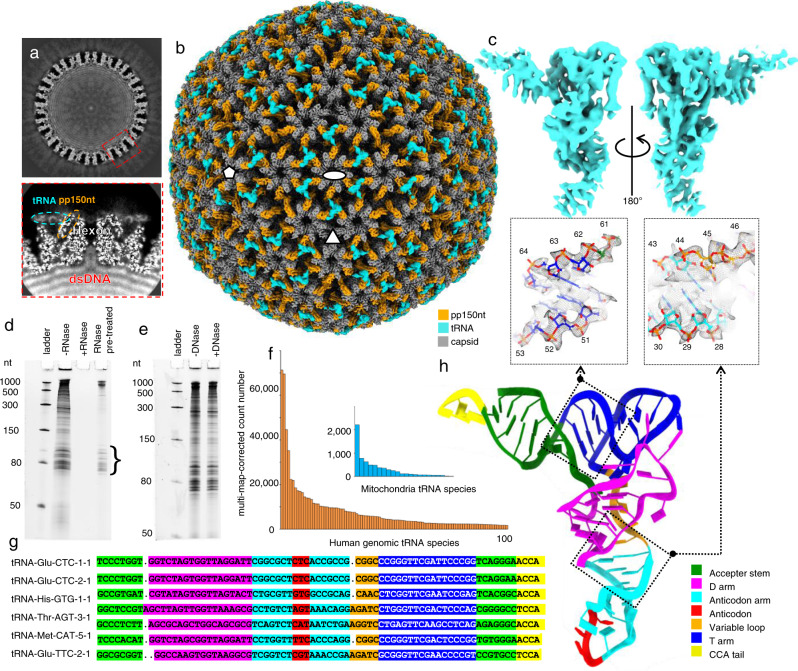
CryoEM reconstruction of HCMV virion and identification of tRNA by sequencing. **a** Central slices of 3D reconstructed HCMV virion (top) and sub-particle reconstruction of the boxed area (bottom) shows the relative locations of dsDNA genome, hexon channel, pp150nt, and L-shaped tRNA densities. **b** Icosahedral reconstruction of HCMV virion. Fivefold, threefold, and twofold axes are denoted by the pentagon, triangle, and oval shapes, respectively. The L-shaped tRNA density (cyan) was resolved. **c** CryoEM structure of L-shaped density. **d**, **e** Analysis of RNA molecules associated with HCMV virus on 15% TBE-urea PAGE. RNA was purified from isolated HCMV virions and separated on electrophoresis as described in the “Methods” section. The curly bracket indicates the size range of RNA that has been subjected to deep sequencing. Infection of MRC-5 cells with HCMV, virion and virion-bound RNA isolation, and electrophoresis analysis was done independently four times (*n* = 4). **f** Multi-map-corrected count number distribution of first 100 abundant human genomic tRNA (orange) and 22 mitochondrial tRNA (blue). **g** Gene sequence of the first 6 abundant human genomic tRNA, colored by the domains of tRNA. **h** de novo atomic model of tRNA-Glu-CTC-1-1 built based on the cryoEM density. Insets show ribbon-and-stick models in mesh density of the boxed regions. dsDNA double-stranded DNA (in a), pp150nt pp150 N-terminal domain (in a and b), nt nucleotides (in d).

We applied icosahedral symmetry-guided, sub-particle reconstruction^8^, and defocus refinement^9^ to obtain sub-particle symmetric and asymmetric (i.e., C1) reconstructions of the HCMV virion around icosahedral 5-fold, 3-fold, and 2-fold axes at up to 2.9 Å resolution (Supplementary Figs. 1 and 2). The overall resolution allows us to see strong amino-acid sidechain densities of the capsid proteins (Supplementary Fig. 3). In addition to protein densities, we also observed that the dsDNA genome is tightly packaged as concentric layers inside the capsid and even protrudes into the confined interior of the hexon channels (Fig. 1a). The DNA confining hexon channel is “secured” from the outside^10^ by β-herpesvirus-specific tegument protein pp150, whose N-terminal one-third (pp150nt) is resolved (Fig. 1a). Attached to these pp150 densities are L-shaped densities (Fig. 1a, b), not well resolved in previous icosahedral reconstruction^10^.

These L-shaped densities bind all over the capsid (Fig. 1b, Supplementary Video 1). Each L-shaped density interacts with three pp150 subunits atop a triplex: one at the corner of the L, and one at each of the two arms of the L. Each arm of the L-shaped density has a 2.3 nm width and has coaxial double helices, which are the defining feature of nucleic acid (Fig. 1c). An overhang at the end of the short arm and a loop at the end of the long arm is reminiscent of the CCA tail and anticodon loop of tRNA, respectively (Fig. 1c, Supplementary Video 2). Indeed, tRNAs have similar L-shaped structures that allow them to fit into the P and A sites of the ribosome^11^.

### Identification of host tRNAs

In order to identify these L-shaped densities, we obtained the primary sequence of these molecules and modeled them into the virion density. To do so, we first purified the virions and treated them with RNase prior to RNA isolation (Fig. 1d “RNase pretreated” lane). This step removed RNA molecules that were non-specifically attached to the virus envelope. The isolated nucleic acids were RNase sensitive (Fig. 1d) and DNase insensitive (Fig. 1e). The Tris-borate-EDTA (TBE) urea polyacrylamide gel electrophoresis (PAGE) of the virion-containing RNA reveals multiple RNA bands whose length spans from 70 to 100 nucleotides (Fig. 1d curly bracket), corresponding to expected sizes of canonical tRNA molecules. This strengthens our interpretation that the L-shaped structure could represent tRNA molecules of variable lengths. We then used RNASeq to perform deep sequencing (Next-generation sequencing, NGS) on purified RNAs whose sizes were between 60 and 100 nucleotides. In order to sequence these RNA molecules, which have stable secondary structures, a library of fragments ranging from 18 to 40 nucleotides was prepared through demethylation and partial hydrolyzation. Sequences of 7,091,354 fragments were generated on an Illumina NextSeq 500 instrument.

Only a negligible amount (0.04% for both strands) of the fragments could be mapped to the HCMV genome, indicating that the majority of RNA molecules isolated from the virions originated from host cells—in this case, the human fibroblast cell used to culture the virus. In contrast, 12.6% of sequenced fragments were mapped to the human tRNA database (Supplementary Data 1), while the rest may be other small non-coding RNAs carried in the virion. The mrcount (multi-map-corrected number of fragments overlapping the tRNA) for each mapped tRNA gene reflects the relative abundance of the tRNA in the sample. The mrcount plot shows that the HCMV has a preference when incorporating different types of tRNAs into its virions (Fig. 1f). For instance, among the 254 mapped genes, the highest mrcount is 71,398 for tRNA-Glu-CTC-1-1 (annotated by GtRNAdb Gene Symbol), while the lowest mrcount is only 6 for tRNA-Leu-CAA-5-1 (Supplementary Data 1). The first 20 tRNA species, all having mrcounts of more than 10,000, contribute to 50% of the total mrcount of mapped human genomic tRNAs. In comparison, although mitochondrial tRNAs share similar sequences and structures with genomic tRNAs in the cytosol, the highest mrcount of mitochondrial tRNAs is 30 times less than that of genomic tRNAs (Fig. 1f, inset). This observation validates the fidelity of our sequencing because mitochondrial tRNA are confined inside the mitochondria and are less likely to be picked up by HCMV.

Because HCMV capsid is assembled in the cellular nucleus, small nucleolar RNAs (SnoRNA) that also form secondary structures and have a similar length to tRNA could potentially be incorporated into the virus capsid. In order to investigate whether the observed density in HCMV virion belongs to SnoRNAs, we performed an additional deep sequencing analysis of RNA samples isolated from HCMV virions targeting SnoRNA. We found that only 0.02% (4296 out of 18,784,576) of all reads were aligned to known human SnoRNA. This finding further supports our conjecture that the observed L-shaped nucleic acid in the HCMV capsid is a tRNA molecule.

### Atomic model of host tRNA and its interactions with tegument protein pp150

The six tRNA genes with mrcount larger than 20,000 are shown in Fig. 1g. Those genes have lengths ranging from 75 to 77 nucleotides, and all have a tRNAscan secondary structure score^12^ of more than 20 bits, showing that they have a stable canonical tRNA structure. Using the sequence of the most abundant tRNA species, we built atomic models into the tRNA density (Fig. 1g, h, Supplementary Video 2). The atomic model consists of four leaflets—acceptor stem (1–8 and 65–71 nt), D-arm (9–26 nt), anticodon arm (27–43 nt), and T-arm (48–64 nt) (Fig. 1g, h). Between the anticodon arm and the T-arm is a 4-nucleotides variable loop (Fig. 1g, h). A CCA tail (72–75 nt) lies at the 5’ end of the structures, enabling the terminal aminoacylation to carry the amino acid for RNA translation. The three-letter anticodon (CTC) is located at the end of the anticodon arm, whose corresponding codon encodes Glutamic acid. The finding that three out of the six most abundant tRNAs HCMV carries encode Glutamic acid, reinforces the idea that HCMV has partial selectivity towards tRNAs.

**Fig. 2:**
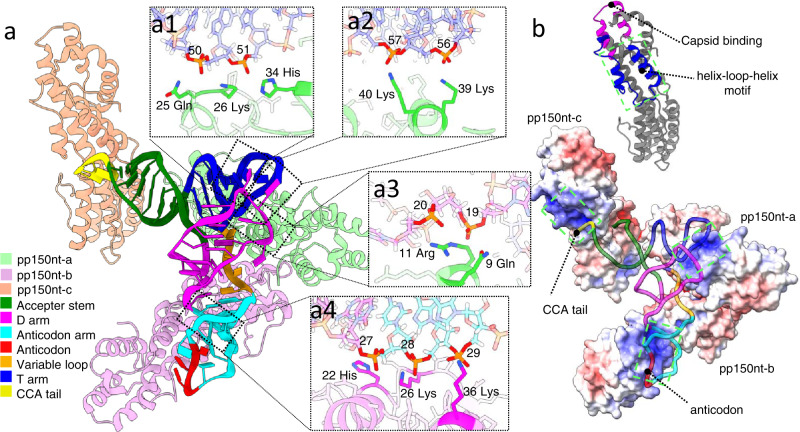
Interaction between tRNA and set-of-three pp150nt. **a** Atomic model of tRNA and its three interacting pp150nts. Insets show the identified tRNA-pp150 interaction sites. **b** Upper panel: helix-loop-helix (blue) and capsid binding (magenta) motifs on a representative pp150nt model. Bottom panel: a pp150nt map surface colored by electrostatic potential superposed with bound tRNA backbone, shown as ribbon.

One tRNA bridges over three pp150 molecules, two of which interact with each other (pp150nt-a and pp150nt-b) while the third is slightly farther apart (pp150nt-c) (Fig. 2a). Four interaction sites between tRNA and pp150s can be identified, all of which might be mediated by electrostatic interactions with the phosphate groups (Fig. 2a, Supplementary Video 2). Among the three, pp150nt-a has the most extensive interactions with the tRNA, binding to both the T-arm (at 25 Gln, 26 Lys and 34 His, 39 Lys, and 40 Lys) and the loop of the D-arm (9 Gln and 11 Arg). pp150nt-b interacts with the phosphate groups in the base-stacked region of the anticodon arm (at 22 His, 26 Lys, and 36 Lys). These interactions only involve the phosphate groups, which introduce less selectivity than base-stacked groups during the recruitment of tRNA, indicating that pp150 could bind to different types of tRNA as shown by the sequencing data (Fig. 1f). Consistent with this interpretation, the phosphate backbone is better resolved than the nucleobases of the tRNA (Fig. 1h, upper insets).

At the two ends of the L-shaped structure, the anticodon and the CCA tail are also located in proximity to pp150, contacting the highly positively charged regions of pp150nt-b and pp150nt-c, respectively (Fig. 2a, b). This positively charged region on pp150nt contains a cluster of positively charged residues on a helix-loop-helix motif (aa 9-43) (Fig. 2b). However, the resolution of the map is insufficient to precisely identify the interaction sites, likely because of the heterogeneity of the bound tRNAs. tRNAs with different lengths should position differently at the two ends and likely interact with different residues inside the positively charged regions of pp150. This adaptability at the anticodon and CCA tail could be a strategy for HCMV to accommodate a variety of tRNAs.

**Fig. 3:**
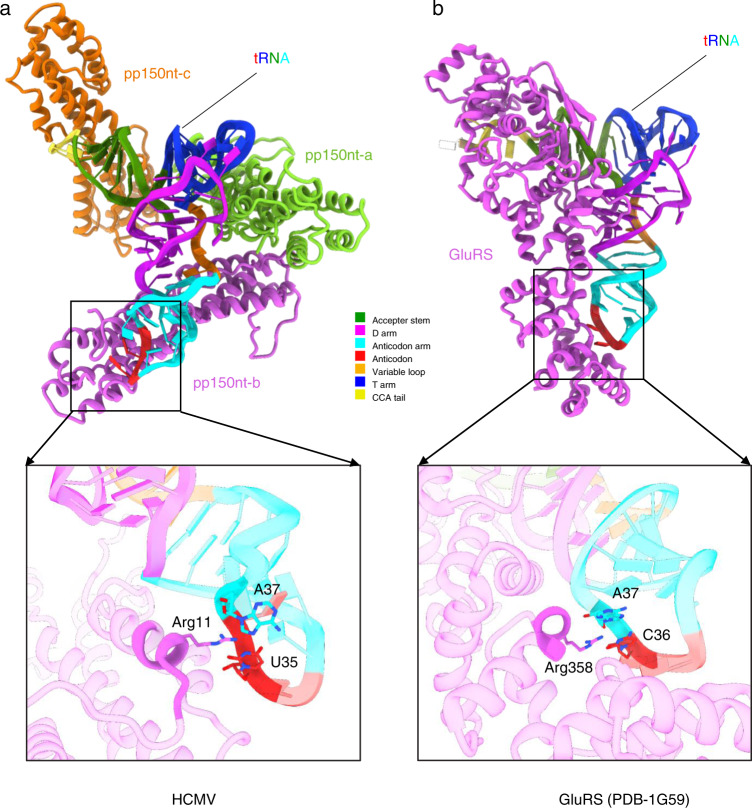
Comparison between the pp150 and glutamyl-tRNA synthetase (GluRS) on their interactions with tRNA anticodon. **a** Atomic model of tRNA and its three interacting pp150nts. Inset shows Arg 11 interaction with the anticodon region of tRNA. **b** Atomic model of GluRS and tRNA. Inset shows Arg358 interaction with anticodon^14^.

The structures of tRNA-Glu and pp150nt presented here, particularly the way of involvement of the anticodon and the CCA tail, are reminiscent of the structures of tRNA-Glu and glutamyl-tRNA synthetase (GluRS)^13,14^. Especially, it was demonstrated previously that the discrimination between the Glu and Gln anticodons (34YUC36 and 34YUG36, respectively) is achieved by a single arginine residue (Arg 358)^14^. The mutation from Arg 358 to Gln 358 resulted in a GluRS that failed to discriminate between the Glu and Gln anticodons. In HCMV, indeed, the Arg 11 of pp150nt-b is proximal to the anticodon of tRNA, resemble Arg 358 previously found in GluRS (Fig. 3). This similarity could explain the partial selectivity towards tRNA-Glu. However, the resolution in the anticodon region of tRNA is relatively low, indicating that the resolved densities possibly represent a mixture of different types of tRNA, thus, the selectively is much lower than that of GluRS.

**Fig. 4:**
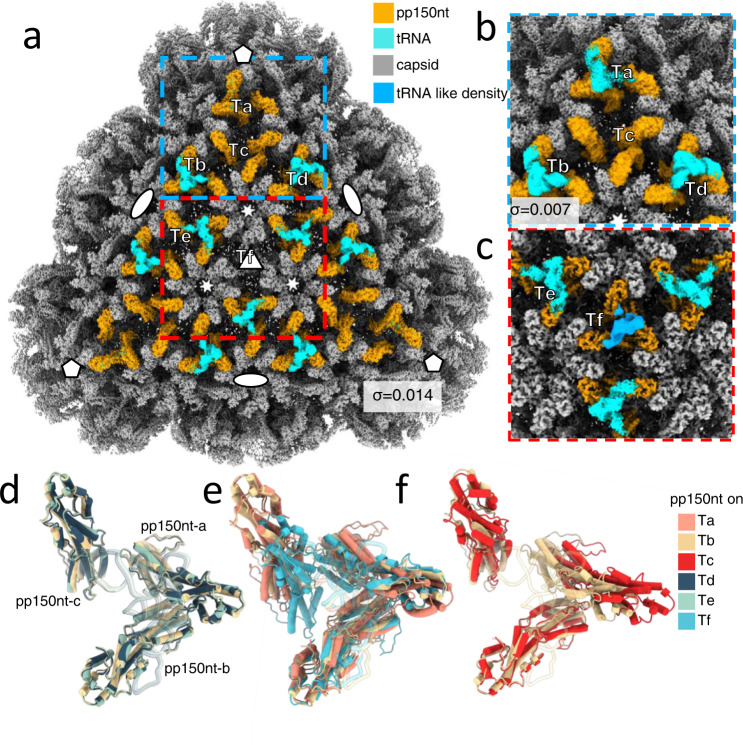
Distribution of tRNA on different triplexes. **a** A facet of the icosahedron of HCMV reconstruction. **b**, Enlarged view of the region in the blue dashed box in **a** but displayed with a lower threshold. Ta’s tRNA-like density becomes visible at a lower threshold. No tRNA binds on Tc. σ represents the threshold used to display 3D structures. **c** Asymmetric reconstruction (e.g., C1) of the region in the red dashed box in **a**. Tf’s tRNA-like density becomes visible at a lower threshold. **d**–**f** Set-of-three pp150 shown as pipes and planks from different triplex areas are superimposed. tRNAs are shown as semi-transparent ribbons. Superimposition among set-of-three pp150 and their corresponding bound tRNA molecules on Tb, Td, and Te (**d**); on Ta, Tb, and Tf (**e**); and on Tb and Tc regions (**f**). The change in pp150 organization affects the ability of tRNA to bind to the pp150.

Each set of three pp150s clusters on one triplex of the capsid^10^. One icosahedral asymmetric unit of the capsid consists of six types of heterotrimeric triplexes—Ta, Tb, Tc, Td, Te, and Tf—located at geographically different local 3-fold positions (Fig. 4a). Consequently, tRNAs can be identified based on the triplexes they reside on. We found that cryoEM densities of tRNAs on the Tb, Td, and Te have the highest resolution, whereas the tRNA on Ta has a much weaker density, not observable at a higher threshold (Fig. 4a, b). Tf and its corresponding pp150s are smeared out by the local 3-fold averaging due to the icosahedral symmetry imposed during previous reconstructions^10^. Using sub-particle classification, we released the 3-fold symmetry and obtained a reconstruction of the asymmetry structure of Tf at 3.6 Å resolution (Supplementary Fig. 4). Like other triplexes, Tf contains one copy of Tri1 protein and two conformers of Tri2 proteins (Supplementary Fig. 4). After this symmetry relaxation, a tRNA-like density along with the underlying three asymmetric pp150s emerged (Fig. 4c). However, this density is weaker than the tRNA density on Ta. Interestingly, the atomic model of Tf-bound pp150-c exhibits some conformational differences from those bound elsewhere (Tb, Td, and Te) (Supplementary Fig. 5a), as does the buttress domain (a.a. 1139–1153) of two of the three major capsid protein (MCP) subunits surrounding Tf (Supplementary Fig. 5b and c). Finally, on the triplex Tc, no tRNA density was observed, even at a low threshold (Fig. 4b).

These differences in the robustness of tRNA densities on different triplexes likely reflect the varying level of flexibility or binding affinity of tRNAs on the underlying set-of-three pp150 molecules. The set-of-three pp150 proteins on Tb, Td, and Te match well, and above those triplexes are the highest density of tRNA (Fig. 4d). A slight change in position of pp150nt-c on Ta and Tf (Fig. 4e), which alters only the flexible interactions between the CCA tail and pp150, dramatically reduces tRNA binding or increases the flexibility of tRNA (Fig. 4b, c). In addition, altering the positions of pp150nt-a on Tc resulted in a complete absence of tRNA density (Fig. 4b, f). The altered pp150 position correlates with the larger distances among Tc-adjacent hexons^15–17^. Thus, pp150s on three out of six types of triplex have a relatively strong density of tRNA, which suggests that, while the pp150-tRNA binding is specific, a particular configuration of the set-of-three pp150 is necessary for optimized or stabilized binding between them.

### Host tRNA-pp150 binding is specific and likely unique to HCMV among herpesviruses

To test whether the above-identified interactions between host tRNA and tegument protein (pp150) are unique to HCMV, we also explored the existence of RNA-like density in cryoEM reconstructions of other herpesviruses. To date, virion structures of seven of the eight human herpesviruses have been determined to near-atomic resolution^10,15,17–22^, so as for two non-human herpesviruses^23,24^. Among them, HCMV, MCMV, and HHV-6 belong to the β-herpesvirus subfamily, which has pp150 (as in HCMV) or a pp150 homolog (as in MCMV and HHV-6) that forms a capsid-associated tegument complex (CATC) emanating from some triplexes. Notably, this pp150-like protein is specific to β-herpesviruses; and members of the α-herpesvirus and γ-herpesvirus subfamilies lack a homolog of pp150. Unlike the trimeric pp150 CATC in HCMV (Figs. 1b and 4), the CATC in MCMV is dimeric^24^, while that in HHV-6 is a complex containing four subunits of the pUL11 tegument protein^15^, the pp150 homolog. We have applied the same sub-particle reconstruction workflow employed above for HCMV to process previous cryoEM images of HHV-6B and MCMV virions. Surprisingly, our sub-particle reconstructions of both MCMV and HHV-6B reveal a lack of tRNA binding to their pp150 homologs, even at a low-density threshold (Supplementary Fig. 6). Consistent with our RNA deep sequencing results, HCMV does not encode viral tRNA based on tRNA gene prediction^12^. By contrast, murine γ-herpesvirus 68 (MHV-68) encodes eight types of viral tRNA (vtRNA) which mimics the features of cellular tRNAs and is incorporated into the virion^3^. Because MHV-68 is a member of the γ-herpesvirus subfamily and does not contain a pp150 homolog, the location of vtRNA in MHV-68 should be different from that of the host tRNA in HCMV. These observations further support that the binding between HCMV pp150 and human tRNA is specific and host tRNA recruitment is likely unique to HCMV among herpesviruses.

## Discussion

We have visualized here RNA molecules in a DNA-genome-containing virus. The only previous example known to have both RNA and DNA visualized is an intermediate prohead capsid of bacteriophage φ29, which uses what is called pRNA to temporarily attach a DNA packaging protein motor to the capsid to assist genome packaging^25,26^. By contrast, the RNA observed here in the DNA virus HCMV is integrated as part of the nucleocapsid. Thus, a virus as a biochemical entity may recognize and package both DNA and RNA, and either of viral or host origin.

The biological relevance of pp150 binding specific tRNAs is currently under investigation, but we propose that packaging host tRNA benefits virus maturation and spread in the following ways. First, the tRNA could function as additional “cement” to withstand the high pressure that comes from accommodating such a large dsDNA genome. Indeed, the HCMV genome is 236 kb, which is larger than those of MHV-68 (118 kb) or HHV-6B (160 kb) and can benefit from capsid encapsulation further stabilized by the bound tRNA. Second, the tRNA pool in an HCMV-infected cell would be highly biased by the tRNA selectivity of HCMV’s pp150. Thus, HCMV would be similar to some RNA viruses, such as human immunodeficiency virus (HIV), which modulate the host cell tRNA pool to improve translation efficiency^27^. Although we are unable to tell from cryoEM density whether the HCMV associated tRNAs are amino-acylated due to the flexibility of the CCA tail, we suspect that both charged and uncharged tRNA are incorporated into HCMV, because the phosphate backbone of the tRNA is primarily responsible for the interactions with pp150. It is likely that HCMV production depletes the charged tRNA of the host, and consequently affects the function of the host cell. Third, tRNAs play versatile roles in cellular processes^28–30^. The packaged RNAs could impart HCMV’s ability to carry information across infected cells by unleashing regulatory tRNAs upon cell entry to modulate functional states of the newly infected cell. For example, recent discoveries have implicated tRNA-derived fragments as small non-coding RNAs involved in epigenetic control of gene expression^31–33^. tRNAs also interact with many proteins that regulate cellular processes^34^, such as apoptosis^35^, cell cycle progression^36^, and anti-viral defenses^37,38^. Therefore, beyond the general significance of demonstrating host RNA inside a DNA virus, the direct visualization of these non-coding RNA now opens the door for a plethora of investigations, from viral-host interactions to anti-viral drug design.

## Methods

### Isolation of HCMV virions

Two batches of isolated HCMV virions were prepared for this paper. The first one was described in our previous paper^10^. In order to improve resolution from our previous results, we isolated more virions for data collection with smaller pixel sizes; the detailed virion purification method is listed below.

Human fibroblast MRC-5 cells (ATCC CCL-171) were seeded on 18 × 175 cm^2^ flasks in Eagle’s Minimum Essential Medium (EMEM; ATCC 30-2003) supplemented with 10% fetal bovine serum (FBS) and 100U/ml of Penicillin-Streptomycin and cultured until they reached ~80–90% confluency, about a week. The cells were then infected with HCMV (AD169 strain, ATCC VR-538). The growth media were replaced with fresh ones 4 days after infection. The media containing secreted HCMV virions were collected at 8 days post-infection and spun an SW28 rotor (Beckman) at 80,000 × *g* for 1 h at 4 °C. The supernatant was discarded and the pellet in each tube was resuspended in 50 µl of cold phosphate-buffered saline (PBS). About 0.7 ml of combined pellet suspension from all ultracentrifuge tubes were loaded on 15–50% linear gradient of sucrose made in PBS and spun in SW41 (Beckman) rotor at 80,000 × *g* for 1 h at 4 °C. About 1 ml of the visible virus-containing band was collected into a new tube and mixed with 10 ml of sterile PBS solution in order to dilute sucrose. The virions were concentrated at 80,000 × *g* for 1 h at 4 °C and resuspended in 0.1 ml of PBS.

The first batch of purification used detergent NP-40 prior to plunging freezing to reduce the size of the virion particle. When we prepared the second batch, the defocus refinement method became available^9^, eliminating the necessity of this detergent treatment. Avoiding detergent treatment also prevents the possible loss of tegument proteins in the second batch of purification.

To prepare EM grids for cryoEM imaging, aliquots of 2 μl of the sample were transferred to Quantifoil grids (2/1). For the first batch of samples, the grids were blotted for 20 s in an FEI vitrobot with 100% humidity and plunged into liquid ethane. For the second batch, the samples were vitrified using a manual plunger with 6 s blot time.

### RNA purification and electrophoresis

Total RNA from virions was isolated with miRNeasy (Qiagen 217004) according to manufacturer instructions. Virions were processed directly with miRNeasy or were pretreated with RNase One (Promega M4261) to hydrolyze RNA molecules outside of the virion. Total RNA was eluted in 50 µl of nuclease-free water and processed with a DNA-free removal kit (Fisher AM1906) to eliminate any residual DNA contamination. RNA amount and quality were assessed using a NanoDrop spectrophotometer based on light absorption at 260 nm wavelength.

The purified RNA molecules were separated on 15% TBE-urea polyacrylamide gel (Novex EC6885BOX). RNA samples were mixed with 2× TBE-urea sample buffer (Novex LC6876), heated to 85 °C for 5 min, and cooled on ice prior to loading on 15% TBE-urea gel. Two hundred and fifty nanograms of low-range ssRNA from NEB (N0364S) were used as the size ladder lane. The gel was stained with SYBR Gold (Invitrogen S11494) for 20 min and visualized with Gel Doc (Bio-Rad).

### tRNA and SnoRNA sequencing

RNA sequencing was performed in ArrayStar company with the Next-Generation Sequencing methods described below.

Total RNA was quantified using a NanoDrop ND-1000 instrument. Three micrograms of total RNA were separated on a urea-polyacrylamide gel, and tRNA was further isolated from a size window of 60–100 nt. These RNAs were then m1A&m3C demethylated and partially hydrolyzed according to the Hydro-tRNAseq method. Partially hydrolyzed and re-phosphorylated tRNA fragments were then converted to small RNA sequencing libraries using NEBNext® Multiplex Small RNA Library Prep Set for Illumina® kit (New England Biolabs). Size selection of ∼140–155 bp PCR amplified fragments (corresponding to ∼19–35 nt tRNA fragments size range) was performed. The subsequent tRNA-seq libraries were qualified and absolutely quantified using Agilent2100 BioAnalyzer. Libraries were equally mixed and sequenced for 50 cycles on Illumina NextSeq 500 system using NextSeq 500/550 High-Output v2 kit (75 cycles) according to the manufacturer’s instructions.

After Illumina quality control was examined using FastQC software (http://www.bioinformatics.babraham.ac.uk/projects/fastqc/), the sequencing reads were 5’, 3’-adaptor trimmed and filtered (length < 18nt or length > 40nt) with cutadapt^39^, and then aligned to cytoplasmic mature-tRNA sequences from GtRNAdb while including the mitochondrial tRNA sequences from mitotRNAdb using BWA^40^ software. For tRNA alignment, the maximum mismatch was two^41^. The statistical information is shown in the table (Supplementary Data 1).

For each tRNA sequence-based profile, the mapped reads number was used to estimate the expression level of each tRNA. The tRNAs expression profiling was calculated based on a multi-map-corrected number of reads overlapping the tRNA (mrcount) which was calculated with the formula:

1 $${{{mrcount}}}=\mathop{\sum }\limits_{i=1}^{n}\frac{1}{{m}_{i}}$$

where *i* represents the *i*-th read, *n* is the number of total reads, and *m*_*i*_ is the number of mapped locations for the *i*-th read aligned to tRNA reference. The information of mrcount for each tRNA reference is shown in the table (Supplementary Data 1).

We also aligned those sequenced fragments to the HCMV genome using ‘water’, the local sequence alignment tool in EMBOSS suite^42^. In order to facilitate analysis, the original file containing 7,091,354 sequences was split into 7091 files, each of which contains about 1,000 reads. The ‘water’ command was then run to compare those files to the HCMV genome with parallel computing. Sequences that matched 100% of the viral DNA were counted, and their amount was expressed as a percentage of total reads.

For SnoRNA, RNA was prepared as above. This preparation was sent to ArrayStar for the library preparation from total RNA (3’- and 5’-adapter ligation; cDNA synthesis; size selection of ~170–370 bp after PCR amplification), SnoRNA sequencing with Illumina technology, and mapping of sequences to the human genome was performed by ArrayStar with a fee for service.

### CryoEM imaging

All cryoEM imaging was performed with an FEI (Thermo-Fisher) Titan Krios electron microscope operated at 300 kV and liquid nitrogen temperature. Movies were recorded using a Gatan K2 Summit direct electron detection camera.

For the first dataset, the cryoEM imaging method was described in^10^. Briefly, the images were acquired using Leginon software^43^ operated in counting mode at a nominal magnification of ×31,120, giving a pixel size of 1.61 Å per pixel. The dose rate of the electron beam was set to ~7 electrons per physical pixel per second on camera, corresponding to a dosage of ~2.7 e–/Å^2^/s. Image stacks were recorded at 4 frames per second for 14 s, and a total of 12,000 movies were ultimately captured.

To further improve the resolution, we obtained a second dataset with a new batch of isolated virions (described above). The images were acquired with a K2 summit camera operated in super-resolution mode and using SerialEM software^44^ at ×105,000 magnification which yields 1.36 Å per physical pixel (0.68 Å per pixel of super-resolution image). The dose rate of the electron beam was set to ~11 electrons per physical pixel per second on camera, corresponding to a dosage of ~5.9 e–/Å^2^/s. Image stacks were recorded at 5 frames per second for 8 s, and a total of 16,522 movies were imaged with a six-day imaging session.

After cryoEM imaging, each movie stack was drift-corrected^45^ and averaged to produce a corresponding micrograph. Defocus values for each micrograph were determined with CTFFIND3^46^. A total of 53,500 virion particles (1024 × 1024 pixels) in the first dataset and 43,666 virion particles (1280 × 1280 pixels) in the second dataset are boxed out from the micrographs.

### Icosahedral reconstruction and sub-particle extraction

We used the protocol for sub-particle extraction and reconstruction established in our previous paper^8^.

First, icosahedral reconstruction with I3 symmetry, in which one of the 5-fold axes is on the *Z*-axis of the reconstruction, was performed using Relion3^9,47^ with two and four times binned particles for the first and the second dataset, respectively. The sub-particle reconstructions around 5-fold, 3-fold, and 2-fold axes were performed for the first dataset. Sub-particle reconstruction around the 2-fold axis was obtained for the second dataset, as the 2-fold axis, sub-particle reconstruction contained better-resolved tRNA densities (i.e., tRNA on Tb, Td, and Te).

To extract 5-fold vertex sub-particles, we expanded the relion data STAR file with I3 symmetry using the Relion ‘relion_particle_symmetry_expand’ command to create a symmetry expanded data STAR file, which contains 60 orientations for each virus particle. We noted that there are five redundant orientations relative to each vertex that differ only in their rot angles (the first angle rotated about the z-axis). Given this observation, we then assigned 60 orientations into 12 groups with 5 orientations in each group. Importantly, the orientations within a group each had different rot angles, but the same tilt and psi angles. Lastly, we selected one orientation in each group as the orientation of a vertex, thereby generating one orientation for each vertex out of the 60 icosahedral-related orientations. Using those orientations, the two-dimensional positions of each sub-particle of vertices were calculated on their respective particle images based on the equations in our previous paper^8^. The sub-particles were extracted with ‘relion_preprocess’ command with a box size of 256 pixels.

To extract sub-particles around 2-fold and 3-fold axes, we modified the orientations in the I3 symmetry expanded data STAR file obtained from the icosahedral reconstruction. To do so, we derived the rotation matrix from the three Euler angles in the star file. This rotation matrix was then multiplied with an additional rotation matrix. The additional rotation matrix was calculated manually so as to rotate the 2-fold and 3-fold axes to the *Z*-axis of the final reconstruction. We then converted the multiplied rotation matrix back to the orientations represented by Euler angles. We subsequently grouped those Euler angles into 30 and 20 groups for sub-particles around 2-fold and 3-fold axes, respectively, based on the fact that the rot angles should be the same in each group. We then selected one orientation in each group as the orientation of a vertex. Using those orientations, the two-dimensional positions of each sub-particle of vertices were calculated on their respective particle images. The sub-particles were extracted with ‘relion_preprocess’ command with 256 and 320 for the first and the second datasets, respectively.

### Sub-particles reconstruction and refinement

Three-dimensional auto-refinement for 2-fold, 3-fold, and 5-fold subparticles was performed with their corresponding symmetry, with only a local search for orientation determination. The references were reconstructed by ‘relion_reconstruct’ command and filtered to 40 Å for each refinement.

To overcome the well-documented depth-of-focus problem^48^ for enormous virus particles, we performed defocus refinement for the subparticles using ‘relion_ctf_refine’^9^ after the first round of auto-refinement. Both per-particle defocus refinement and beam tilt were estimated in this step. The resulting new STAR files were subjected to the second round of auto-refinement. For the first dataset, 1,604,850; 1,069,900 and 529,860 sub-particles were used for the refinement of reconstructions around 2-fold, 3-fold and 5-fold axes, respectively. For the second dataset, 1,057,703 sub-particles were used for the refinement of reconstructions around a 2-fold axis.

The final resolution of the reconstruction was estimated with two independently refined maps from halves of the dataset with gold-standard Fourier shell correlation (FSC) at the 0.143 criterion^49^ using ‘relion_postprocess’ and determined to be 3.2 Å for all 2-fold, 3-fold, and 5-fold sub-particle reconstruction for the first dataset (Fig. S2, a–c). The resolution of the 2-fold sub-particle reconstruction for the second dataset was determined to be 2.9 Å (Fig. S2d).

To improve the quality of the map of tRNA, we averaged the two reconstructions around the two-fold axis from the two datasets by “fit in map” and “volume add” functions in UCSF Chimera^50^. Because all tRNAs densities on the Tb, Td, and Te regions have good quality, we further averaged densities encompassing the regions tRNA and pp150s on those triplexes, generating the map for atomic model building.

### Reconstruction of the sub-particles around 3-fold axes without symmetry

Due to the three-fold averaging of the 3-fold sub-particle reconstruction, triplex Tf and its corresponding pp150s are smeared out. Thus, to better resolve the tRNA density residing on the Tf, we sought to obtain the sub-particle reconstruction without symmetry, using the methods described in our previous paper^18^.

To do so, we used the first dataset and did 3D classification of the sub-particles with 3-fold symmetry to exclude the DNA-devoid particles that may affect further symmetry relaxation. This classification was done without orientational search and limited the resolution in classification to 20 Å, which is similar to the diameter of double-stranded DNA. After 50 iterations, four classes were generated. Two of the classes show dsDNA genomic density. We selected the one best class containing 253,399 sub-particles and relaxed the 3-fold symmetry with ‘relion_particle_symmetry_expand’, and then did a second round of sub-particle classification without symmetry. The second round of classification was also performed without orientational search and generated three classes that are similar in structure but have a rotational difference of approximately 120° in between classes. We selected particles in one best class and performed 3D refinement with local orientational search, yielding asymmetric sub-particle reconstruction. A total of 269,700 sub-particles were used in the refinement. The final resolution of the asymmetric reconstruction was determined to be 3.6 Å with gold-standard FSC (Supplementary Table 1).

### Atomic modeling, model refinement, and graphics visualization

For the atomic model of tRNA, we first generated the model of tRNA with its sequence using RNAComposer^51^. We then refined the model using the PHENIX real-space refine function^52^. The real-space refinement was performed for six rounds with global minimization, morphing, and simulated annealing until the correlation between the map and the model stopped improving. The models of this refined tRNA and pp150nts from previously published HCMV models^10^ were docked into the Tb, Td, and Te regions of 2-fold sub-particle reconstruction, and then all side chains were manually adjusted into the density map. These models were then subjected to real-space refinement against the 2-fold sub-particle reconstruction using PHENIX. We then again manually corrected rotamer, plane, and bond/angle outliers using Coot^53^ by referring rotamer and geometry analyses in Coot, and the overall structural assessment report generated by the wwPDB validation web server^54^. The real-space refinement and manual structural correction were conducted for two rounds until the final structure showed no improvement.

The models of triplex and three pp150nts of Td region, C3 MCP and C3 SCP from previously published HCMV models^10^ were docked in the Tf region of the C1 3-fold sub-particle reconstruction. With the same strategy as 2-fold sub-particle models, two rounds of iterative refinement were carried out to obtain final improved models (Supplementary Table 1).

Visualizations of the atomic model, including figures and movies, are made by UCSF ChimeraX and Chimera^50,55^.

### Reporting summary

Further information on research design is available in the Nature Research Reporting Summary linked to this article.

## Supplementary information

Supplementary Information

Supplementary Video 1

Supplementary Video 2

Description of additional supplementary files

Reporting Summary

## Data Availability

The four cryoEM maps have been deposited in the Electron Microscopy Data Bank (EMDB) under accession numbers EMD-23376 [5-fold reconstruction], EMD-23377 [3-fold reconstruction], EMD-23386 [3-fold reconstruction without symmetry], EMD-23388 [2-fold reconstruction]. The atomic models of pp150 and tRNA [Tb, Td, and Te, related to EMD-23388], and models of asymmetrical Tf region (related to EMD-23386) have been deposited in the Protein Data Bank under accession numbers 7LJ3 and 7LIV, respectively. The deep sequencing data have been deposited to Gene Expression Omnibus under accession numbers: tRNA-GSE167037 and snoRNA-GSE168263. Source data of RNA sequencing for Fig. 1 is provided with this paper. Source data are provided with this paper.

## Source data

source data
