# Supplementary material for: Structure of human cytomegalovirus virion reveals host tRNA binding to capsid-associated tegument protein pp150
Source: Nat Commun. 2021 Sep 17;12:5513. doi: 10.1038/s41467-021-25791-1 (PMC8448752; doi:10.1038/s41467-021-25791-1)
Supplement: Supplementary file 1 — Supplementary Information [file 41467_2021_25791_MOESM1_ESM.pdf]

Structure of human cytomegalovirus virion reveals host tRNA binding to capsid-associated tegument protein pp150

Yun-Tao Liu et al.

Supplementary Table 1 Cryo-EM data collection, refinement and validation statistics

	C5 5f sub-particle reconstruction (EMD-23376)	C3 3f sub-particle reconstruction (EMD-23377)	C1 3f sub-particle reconstruction (EMD-23386) (PDB-7LIV)	C2 2f sub-particle reconstruction (EMD-23388) (PDB-7LJ3)
**Data collection and processing**				
Magnification	31,120×	31,120×	31,120×	31,120×
Voltage (kV)	300	300	300	300
Electron exposure (e–/Å^2)	37.8	37.8	37.8	47.2
Defocus range (μm)	1.0-3.0	1.0-3.0	1.0-3.0	1.0-3.0
Pixel size (Å)	1.61	1.61	1.61	1.36
Symmetry imposed	C5	C3	C1	C2
Initial particle images (no.)				
Final particle images (no.)	529,860	1,069,900	1,069,900	1,604,850
Map resolution (Å)	3.2	3.2	3.6	2.9
FSC threshold	0.143	0.143	0.143	0.143
Map resolution range (Å)				
**Refinement**				
Initial model used (PDB code)			pdb:5vku	pdb:5vku
Model resolution (Å)			3.5/3.7	3.9
FSC threshold			0.143/0.5	0.143
Model resolution range (Å)				
Map sharpening *B* factor (Å^2)			-119	-124.9
Model composition				
Non-hydrogen atoms			12	12
Protein residues			47058	25714
Ligands			5903	2564
B factors (Å^2)				
Protein				
Ligand				
R.m.s. deviations				
Bond lengths (Å)			0.009	0.005
Bond angles (°)			0.9	0.7
Validation				
MolProbity score			2.6	2.6
Clashscore			26.4	26.4
Poor rotamers (%)			3	3.1
Ramachandran plot				
Favored (%)			94.5	96
Allowed (%)			4.9	3.6
Disallowed (%)			0.6	0.4

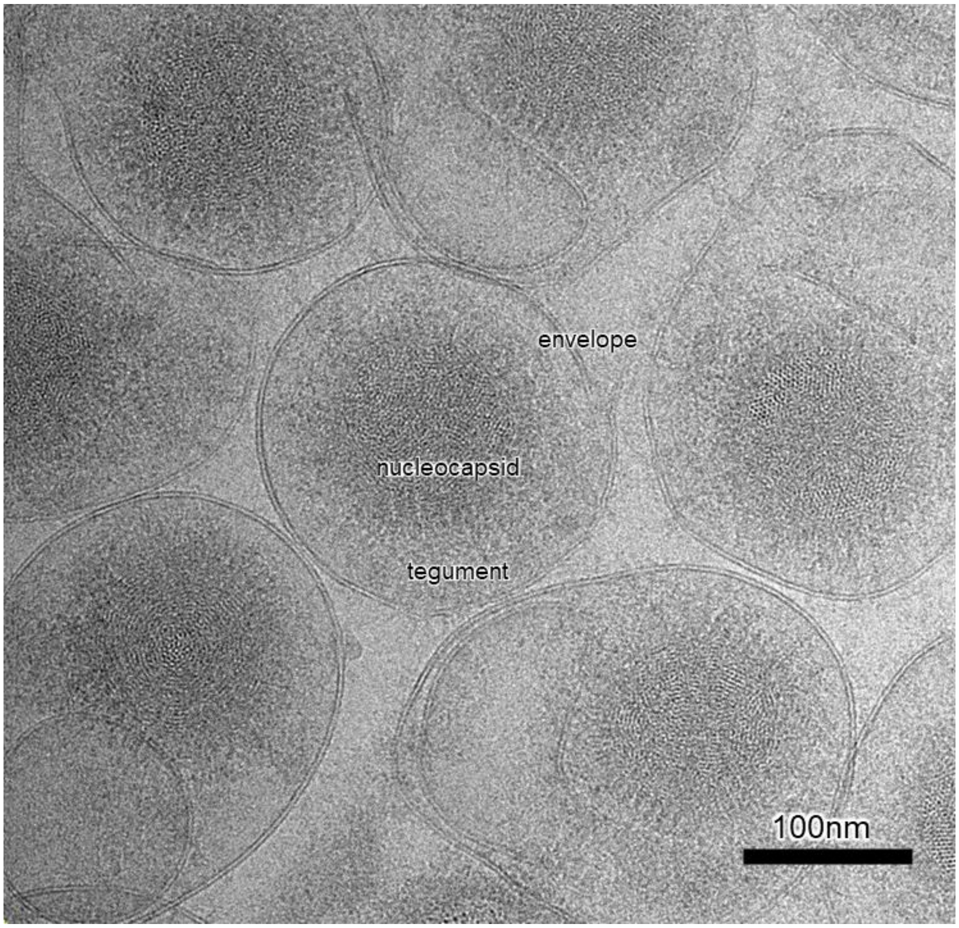

Supplementary Fig. 1 A representative cryoEM micrograph of the HCMV virion. The virion sample was vitrified in liquid ethane on Quantifoil grid using manual plunger. The grid was loaded into Titan Krios microscope operated under 300 kV accelerating voltage. The imaging conditions were set to 105,000 × magnification, -1.5 μm defocus and dosage of 5.9 e^-/$Å^2$/sec.

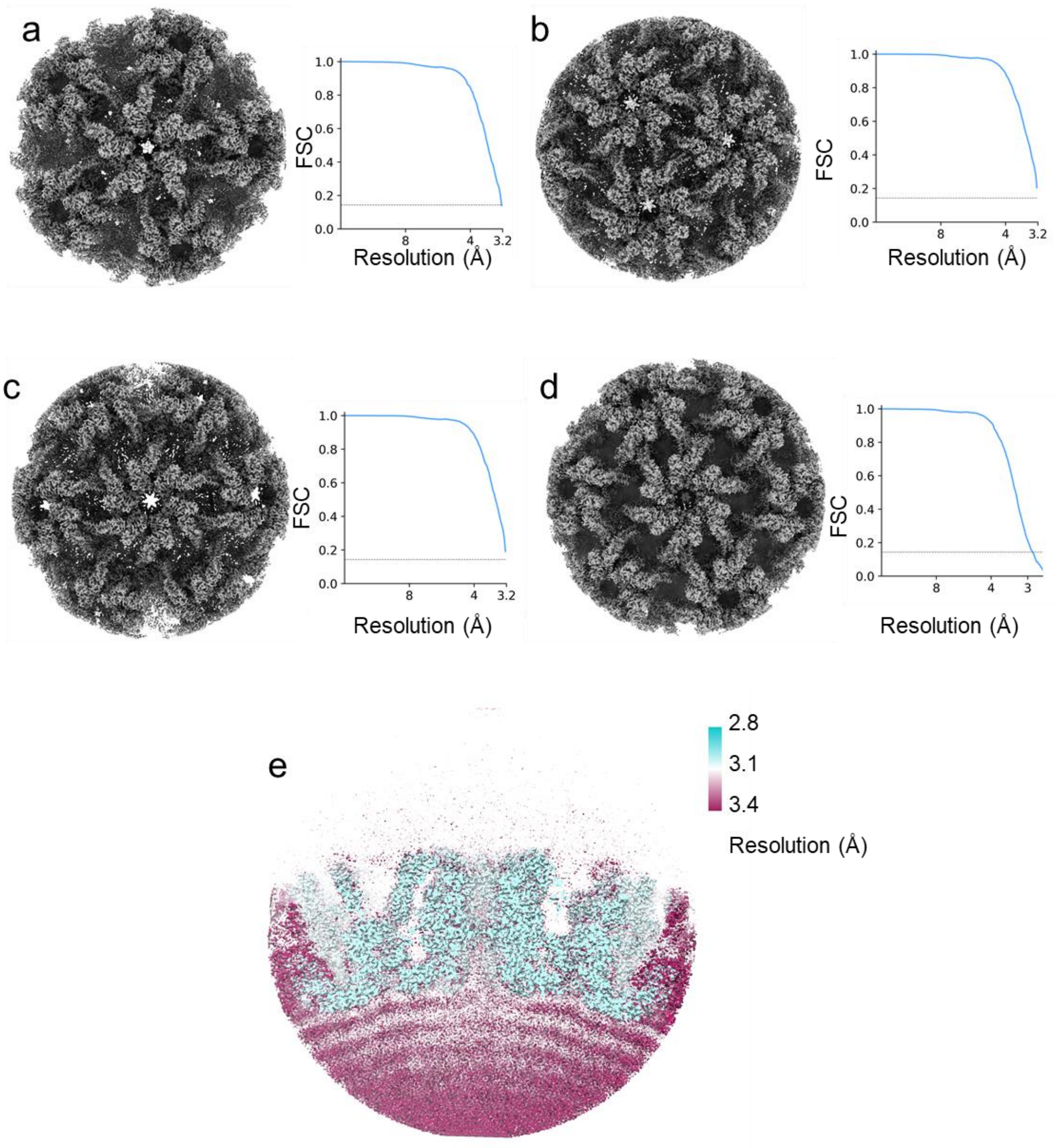

Supplementary Fig. 2 Sub-particle CryoEM reconstruction of the HCMV virion. a-d, Left, 3D view of sub-particle reconstruction of areas around 5-fold axis **(a)**, 3-fold axis **(b)**, 2-fold axis **(c)**, 2-fold axis for the second dataset with smaller pixel size **(d)**. Right, Fourier shell curve (FSC) of the corresponding reconstruction. Dashed lines represent FSC=0.143. **e,** Cut-through view of local resolution map of the 3D reconstruction shown in **(d)**.

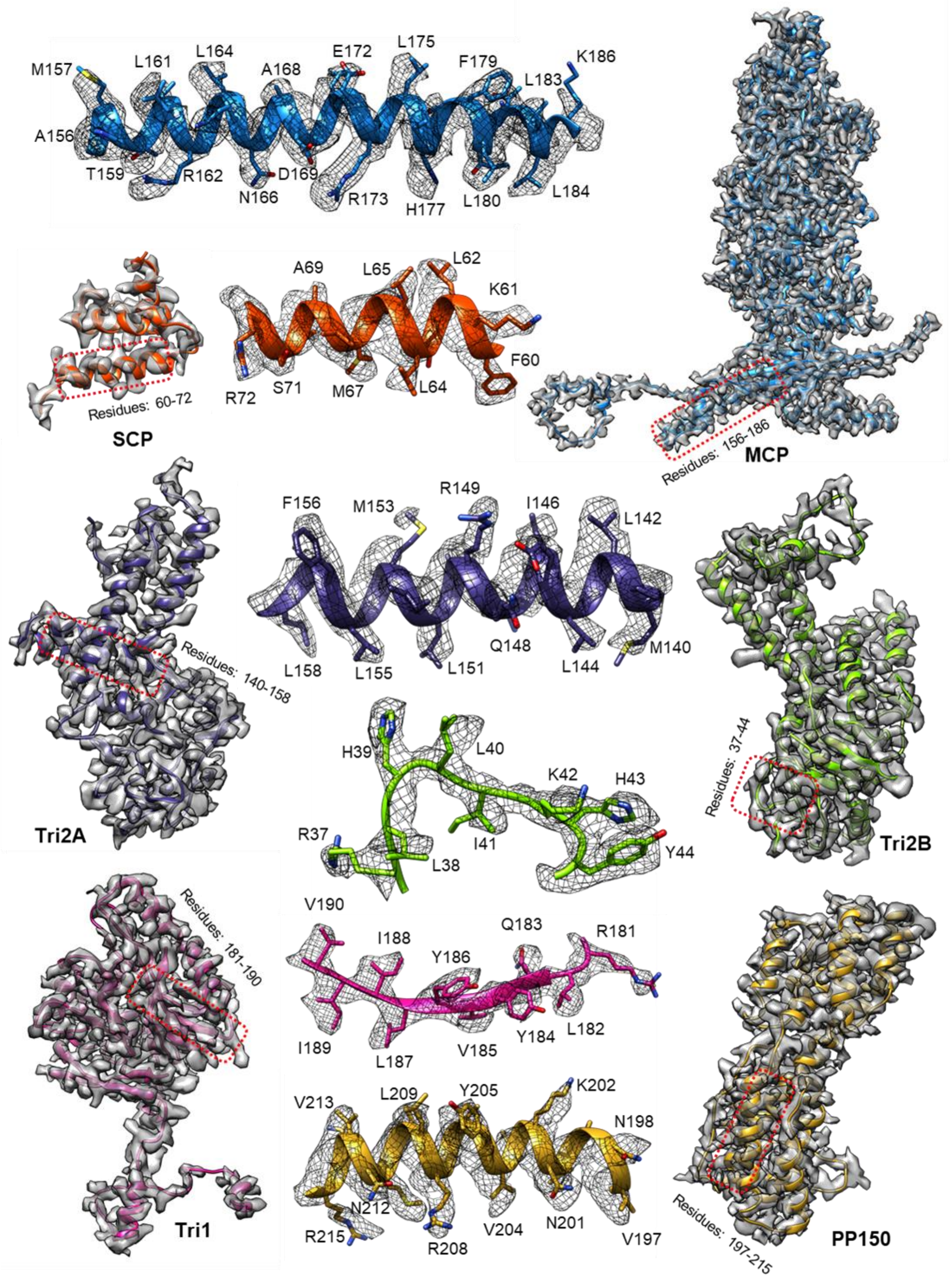

Supplementary Fig. 3 Overall structural and side chain fitting of HCMV capsid and pp150 proteins segmented out from the 2.9Å reconstruction. SCP – small capsid protein, MCP – major capsid protein, Tri2A,B – copies of triplex protein 2, Tri1 – triplex protein 1.

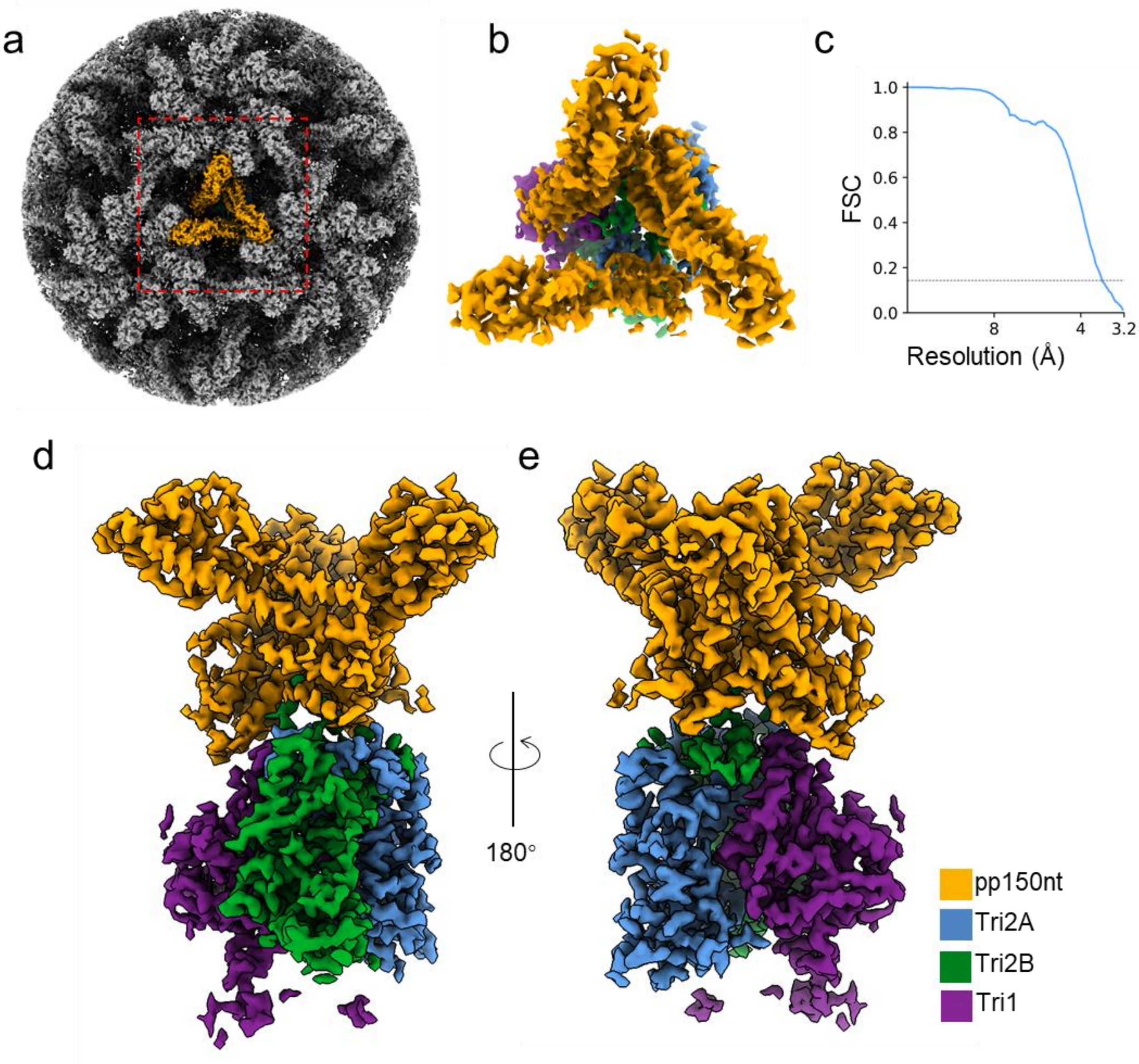

Supplementary Fig. 4 Symmetry relaxed sub-particle reconstruction of the region around triplex Tf. a, Reconstruction of the region around Tf without symmetry. **b,** The Zoomed-in view of the boxed region in **(a)**, but only triplex Tf and the set-of-three pp150 are shown. **c,** Fourier shell correlation curve of the Tf sub-particle reconstruction without symmetry. **d-e,** Side views of the reconstruction shown in **(b)**.

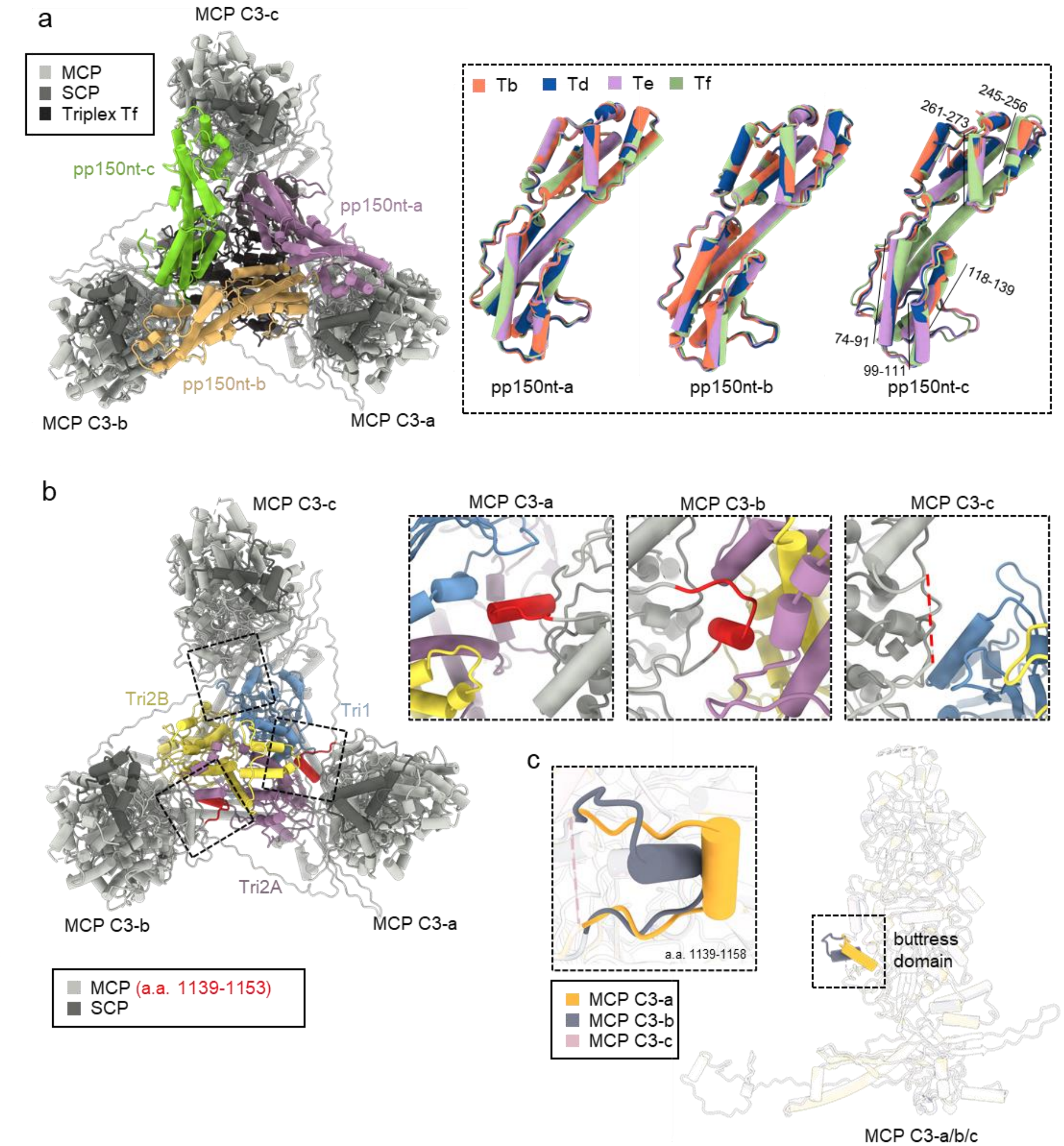

Supplementary Fig. 5 Structures of pp150nt molecules and MCP-triplex interactions in the Tf region. a, Structures of the three pp150nt subunits bound to triplex Tf. Three pp150nt molecules stand against three neighboring MCP molecules in a more compact fashion (left panel) than those bound to triplex Tb. Subunit pp150nt-c on Tf exhibits larger structural difference from those on Tb, Td, and Te, than among pp150nt-a and pp150nt-b subunits (right

panel). **b-c,** MCP-triplex interactions in the Tf region. The heteromeric interactions between the upmost region of triplex Tf and the buttress domain of three neighboring MCP molecules are highlighted in red color **(b)** and detailed in the insets of the three boxed areas. The superposition of the three MCP structures shows that their corresponding fragments of a.a. 1139-1149 (boxed region containing a helix) located in their buttress domains adopt different conformations **(c)**.

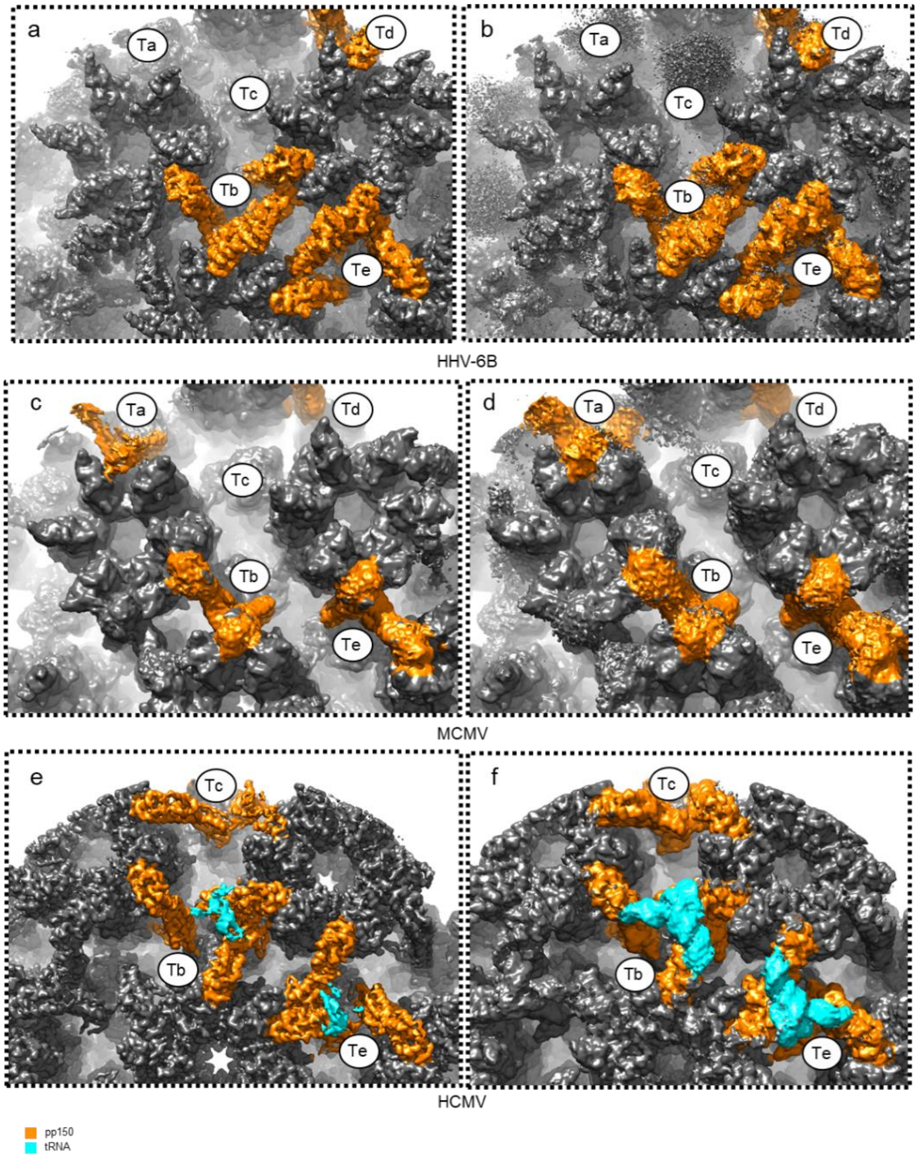

Supplementary Fig. 6 Absence of the tRNA density on the capsid of HCMV and HHV6. a, c and e, Sub-particle reconstruction of HHV-6B (**a**), MCMV (**c**) and HCMV (**e**), respectively. **b, d and f,** Sub-particle reconstruction of HHV-6B (**b**), MCMV (**d**) and HCMV (**f**) at low threshold, respectively.
