# Supplementary material for: Structure of human cytomegalovirus virion reveals host tRNA binding to capsid-associated tegument protein pp150
Source: Nat Commun. 2021 Sep 17;12:5513. doi: 10.1038/s41467-021-25791-1 (PMC8448752; doi:10.1038/s41467-021-25791-1)
Supplement: Supplementary file 4 — Description of additional supplementary files [file 41467_2021_25791_MOESM4_ESM.docx]

Description of additional supplementary files

Title: Supplementary Video 1.

Description: Overall reconstruction of the HCMV virion showing tRNA densities (cyan) bound to the pp150 tegument proteins (orange), which in turn bind to the exterior of the nucleocapsid. Related to Fig. 1b.

Title: Supplementary Video 2.

Description: This movie shows density map and atomic model of tRNA bound to the pp150 tegument proteins inside HCMV, and the interaction sites between pp150 and tRNA. Related to Fig. 1, c and h; Fig. 2a, and Fig. 4, a-c.

Title: Supplementary Table 1.

Description: Cryo-EM data collection, processing, model refinement and validation statistics.
